# Plasma Fabrication and SERS Functionality of Gold Crowned Silicon Submicrometer Pillars

**DOI:** 10.3390/ma13051244

**Published:** 2020-03-10

**Authors:** Paola Pellacani, Carlo Morasso, Silvia Picciolini, Dario Gallach, Lucia Fornasari, Franco Marabelli, Miguel Manso Silvan

**Affiliations:** 1 Departamento de Física Aplicada and Instituto de Ciencia de Materiales Nicolás Cabrera, Universidad Autónoma de Madrid, 28049 Madrid, Spain; paola.pellacani@plasmore.com (P.P.); or; 2 Plasmore S.r.l., Via Vittorio Emanuele II 4, 27100 Pavia, Italy; lucia.fornasari@plasmore.com; 3 Istituti Clinici Scientifici Maugeri IRCCS, via Maugeri 4, 27100 Pavia, Italy; carlo.morasso@icsmaugeri.it; 4 IRCCS Fondazione Don Carlo Gnocchi, Via Capecelatro 66, 20148 Milano, Italy; spicciolini@dongnocchi.it; 5 Departamento de Ciencia, Computación y Tecnología, Universidad Europea de Madrid, 28670 Villaviciosa de Odón, Madrid, Spain; 6 Physics Department, University of Pavia. via A.Bassi, 6. I-27100, Pavia, Italy; franco.marabelli@unipv.it

**Keywords:** SERS, Si pillars, reactive ion etching, gold nanostructures, sputtering, XAS

## Abstract

Sequential plasma processes combined with specific lithographic methods allow for the fabrication of advanced material structures. In the present work, we used self-assembled colloidal monolayers as lithographic structures for the conformation of ordered Si submicrometer pillars by reactive ion etching. We explored different discharge conditions to optimize the Si pillar geometry. Selected structures were further decorated with gold by conventional sputtering, prior to colloidal monolayer lift-off. The resulting structures consist of a gold crown, that is, a cylindrical coating on the edge of the Si pillar and a cavity on top. We analysed the Au structures in terms of electronic properties by using X-ray absorption spectroscopy (XAS) prior to and after post-processing with thermal annealing at 300 °C and/or interaction with a gold etchant solution (KI). The angular dependent analysis of the plasmonic properties was studied with Fourier transformed UV-vis measurements. Certain conditions were selected to perform a surface enhanced Raman spectroscopy (SERS) evaluation of these platforms with two model dyes, prior to confirming the potential interest for a well-resolved analysis of filtered blood plasma.

## 1. Introduction

Surface engineering techniques are increasingly spanning into the field of optics, allowing the design of intricate material configurations with photonic [1], luminescent [2], photochemical [3], plasmonic [4], photovoltaic [5], or even dual [6] properties. The engineering of these structures requires a sequential fabrication in which, most often, a plasma-processing step is involved. The plasma surface engineering of functional structures has in fact found applications in many fields such as biomedicine (both for diagnostics [7,8,9] and therapeutic approaches [10,11,12,13,14]), energy conversion [15,16,17], and the environment [18,19]. The relevance of the plasma processes for the surface engineering of optical structures stems from its versatility to work in deposition (additive process) or etching (removal process) conditions at a wide range of pressures, gas combinations, and temperatures, making the processing of organic and inorganic structures compatible. This versatility of operation in extremely different conditions, and flexibility for the processing of materials of different natures, is scarcely provided by pure ion, electron, or photon techniques. 

The design of plasmonic and other co-allied materials, such as metamaterials, implies not only particular electronic properties provided by the integrated elements or molecules, but also a precise geometric distribution of these materials that define the final properties and applications of the system. For instance, metallic thin films exhibit surface plasmon polaritons (SPPs), i.e., resonant electromagnetic waves traveling on top of metal thin films upon convenient coupling. Most of these metallic structures are processed by magnetron sputtering on glass prisms that facilitate the optical coupling and surface plasmon resonance biosensing applications (the so-called Kretschmann configuration) [20,21]. However, an increase in sensitivity has been achieved by introducing metallic film discontinuities and plasmon blind microdomains, which can be also processed by plasma techniques. In fact, both metallic islands and cavity structures in metals exhibit localized surface plasmons (LSPs) upon appropriate light excitation, a second type of plasmonic wave with much higher sensitivity in view of the extension of the electromagnetic field into the surrounding environment. There exists, thus, many reports on the plasma sputtering processing of discontinuous metallic thin films exhibiting LSPs due to the presence of islands [22,23] and/or cavities [6,24]. In the formation of micro-array technologies, the plasmonic surfaces are complemented with dielectric materials that incorporate a surface antifouling behavior, which provide a contrast during imaging acquisition [25,26]. On the other hand, the formation of Ag gratings has been used to locally densify plasma discharges as a result of the dissipation of the polaritons in the plasma [27]. 

The particular case of biomolecular detection/analysis through surface enhanced Raman scattering requires a particular attention. This technique exploits the notable increase in the Raman active band intensity of biomolecules induced by the presence of strong neighboring electromagnetic fields, which can be induced by plasmonic materials, whether free nanoparticles [28,29], or supported structures [24,30]. Limitations to the amplification of the Raman signal stem however, from its non-proportionality to the different bands, can hinder the identification of the target biomolecule in complex (real) samples [31]. Additionally, undesired chemical reactions can be induced by plasmonic catalytic effects, which can reversibly [32] or irreversibly [33,34] affect the molecular structure, thus hindering the characteristic spectral features of the molecule. 

In light of the aspects mentioned above, the surface engineering of plasmonic structures for Surface Enhanced Raman Spectroscopy (SERS) could be oriented to: a) increase the sensitivity (enhancement factor) of the detection/analysis of specific molecules, b) couple the highest signal enhancement with the most characteristic Raman active bands of a target biomolecule, and c) prevent the plasmon activated catalytic activity of Au surfaces leading to unexpected reactivity. In the present work, we aim to fabricate dual photonic–plasmonic structures consisting of Si pillars partially coated with Au. In particular, we aim to define the experimental parameters that allow the establishment of the relevant wavelength range of operation in the structures when applied in SERS. For the fabrication, we relied on a submicroscale lithographic technique, such as colloidal self-assembly [35,36] through a Langmuir–Blodgett transfer, leading to multidomain hexagonal closed packed monolayers with large areas (cm^2^). The plasma processing steps include the reactive ion etching (RIE) of Si through the colloidal monolayers and the plasma sputtering deposition. RIE is an extremely versatile technique for the micro and nanoscale definition of devices exhibiting photonic [37], plasmonic [38], or even dual (photonic and fluorescent [39], photonic and plasmonic [6]) optical properties. On the other hand, plasma sputtering is considered the most versatile technique for the deposition of metallic thin films (elemental metals [40], ceramic nitride metals [41], metal-organic composites [42,43], and transparent conductive oxides [44]), being crucial for the activation of a wide spectrum of optical devices. 

## 2. Materials and Methods 

**Figure 1:**
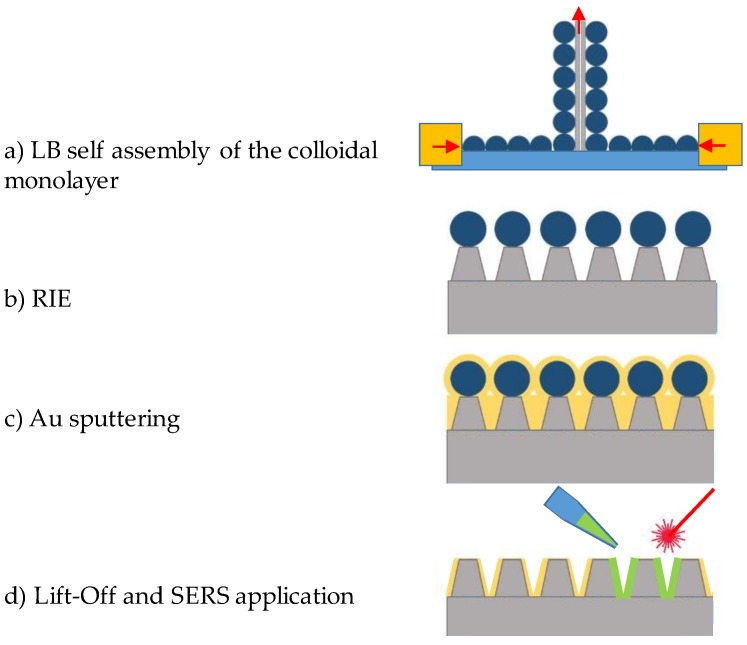
Schematic of the fabrication and final application of the Au crowned Si pillars. (**a**) Langmuir–Blodgett (LB) transfer of the colloidal monolayer, (**b**) reactive ion etching (RIE), (**c**) plasma sputtering of Au, and (**d**) lift-off and surface enhanced Raman spectroscopy (SERS) application.

The entire rationale of our work is depicted in Figure 1, which includes all the experimental steps required to fabricate the gold crowned Si pillars (Au/SiΠ) for SERS bioanalytical purposes; the self-assembly of a colloidal monolayer (**a**), its passing through the reactive ion etching (**b**) and Au plasma sputtering (**c**), to the lift-off of the resistant residues and application in SERS (**d**). The Au/SiΠs were processed on single side polished Si <100> wafers. The substrates were cut in 2 × 2.5 cm^2^ pieces and were mounted in couples with their unpolished side facing each other on the dipping arm of a Langmuir–Blodgett self-assembly tool (KSV, Espoo, Finland) [45]. The substrates were immersed in the subphase (water) prior to the formation of monolayers of polystyrene (PS) spheres (monodisperse, 500 nm diameter). Circa 300 µL of the colloidal solution was re-dispersed onto the subphase surface, ensuring that no drastic increase was induced in the surface tension, revealed by a Wilhelmy plate. Two lateral barriers compressed the colloids, inducing a linear increase of the surface tension, followed by a sharp increase upon conformation of a crystalline monolayer. The transfer step was then initiated by keeping the surface tension constant while simultaneously retrieving the Si substrates vertically. Upon completion of the transfer, the substrates with colloidal monolayers were dried in air and cut in 1 × 1 cm^2^ pieces. 

The RIE was performed in a capacitively charged system (Mini-Lab Plasma-Pod RIE) with 100 cm^2^ stainless-steel plates, an RF power of 60–75 W, a CF_4_:Ar ratio of 5:1, and a total pressure of 3.33 Pa. The objective was to induce a selective ion etching of the substrate, but collateral etching of the mask was noticeable, limiting the maximum exposure time due to colloidal mask degradation (tilting, melting, and total etching, depending on the discharge parameters). The study of the effect of Au deposition and post-processing was performed on flat Si substrates and flat glass coverslides by direct formation of Au nanocavities (i.e., no Si pillar). For this, the same colloidal masks were used, but CF_4_ was substituted for O_2_ to affect the PS colloids without any noticeable etching of the Si substrate. The Au sputtering was performed in a direct current (DC) plasma metallization unit reaching an initial base pressure of 0.5 Pa. The Ar flow was used to regulate the working pressure at 10 Pa with a discharge current of 20 mA. To ensure there was a continuous film over the irregular pillar walls, the process was prolonged for 280 s. After the Au deposition, different conditions were explored to improve the gold microstructure. We first considered a soft annealing at 300 °C for 1 h. Then, we considered a soft Au etching process performed for 1 min in KI (Sigma Aldrich) diluted to 25% v/v in milliQ water. 

For the characterization of the structures, field emission scanning electron microscopy was performed in a XL40 FEG field emission system operated at 10 kV using its through-the-lens (TLD) detector (FEI, Hillsboro, OR, USA). X-ray absorption spectroscopy (XAS) was used to study the local structure of the Au films using the L3-edge (11.9187 keV). The experiments were performed on the BM-25A beamline (5–35 keV) at the European Synchrotron Radiation Facility (ESRF, Grenoble, France). The fluorescence signal was registered with a 13-element Si(Li) detector from an e2v scientific instrument (Chelmsford, England) with the sample placed at 45° with respect to the incident beam. During the measurements, a metallic thin film was used to set and calibrate the energy incoming from the monochromator. The spectra were processed and analysed in terms of radial distribution functions using the Demeter software pack (v0.9.20) [46]. 

Optical measurements in reflectance mode were carried out in two different systems. For the identification of the best performing Au coating, the measurements were performed by coupling through the optical fiber with a UV-Vis spectrometer (SM242, Spectral Products, Putnam, CT, USA) to a microscope objective in normal configuration with the sample surface, and registering the spectrum in the 400–1000 nm range. For the characterization of the full Au/SiΠ structures, transverse magnetic (TM) and transverse electric (TE) polarized light was used in a commercial Fourier transform spectrometer (FT-66, Bruker) equipped with a halogen lamp. The system integrates a home-made variable angle micro-reflectometer. The detection was performed through Si or InSb photodetectors, allowing for measurement on a wide spectral range (500–3300 nm). 

Raman spectra were recorded using an Aramis micro-Raman from Horiba Jobin Yvon (Villeneuve d’Ascq; France) equipped with laser light sources operating at 533, 633, and 785 nm. The Raman spectrometer was calibrated daily using different bands of cyclohexane (i.e., 801.3, 1266.4, and 1444.4 cm^−1^). SERS experiments on Malachite Green (MG) and Rhodamine 6G (R6G) were performed by dropping a small amount (4 uL) of the molecule of interest in water solution (at the specified concentration) on the Au/SiΠ surface. All spectra were acquired after letting the solution dry at room temperature. Spectra were collected using a 50× objective with a 1 s acquisition time. The shown spectra are the average of 50 different acquisitions after baseline subtraction. Finally, SERS spectra were also collected from a drop of human blood plasma previously filtered with three different cut-off molecular weights: 3 KDa, 10 KDa, and 30KDa.

## 3. Results and Discussion

The sequential process of formation and post-processing of the Au/SiΠ structures was analysed prior to the determination of their optical properties and their potential for SERS applications.

### 3.1. Si Pillar Geometry

**Figure 2:**
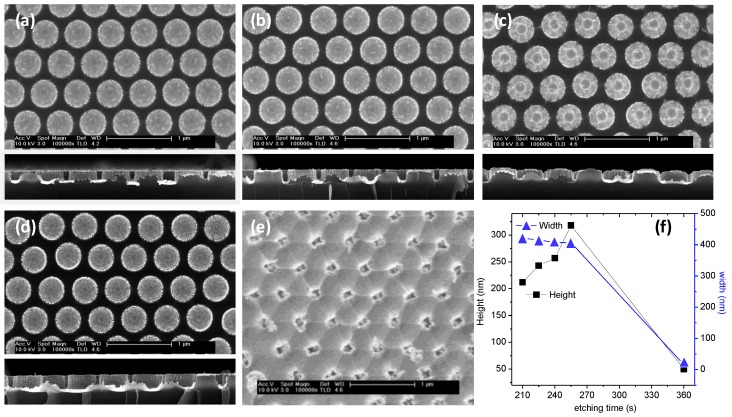
FESEM images of the Si pillar structures obtained after RIE processing during 210, 225, 240, 255, and 360 s (**a**–**e**, respectively) and corresponding cross section view (bottom, except for **e**) obtained after Au deposition to refine image acquisition. **f**) Plot of the evolution of height and width of the Si pillars as derived from the FESEM images.

RIE was selected as an effective process to selectively etch Si in a CF_4_ plasma without drastically affecting the mask. However, the first screening of plasma parameters was performed in view of the special characteristics of the colloidal monolayer masks. In fact, the PS colloids were not dense but rather considerably soft and made contact to the surface only by an interfacial vortex, which limits their resistance to the plasma process and the derived thermal exposure. The RIE process was thus limited to a power of 60 W with a standard gas composition for Si etching, paying special attention to the plasma exposure time. Figure 1a–d shows the surface and lateral field emission scanning electron microscopy (FESEM) view of the Si pillar structures obtained by RIE 210 to 360 s after lift-off of the colloidal mask. For clarity, the cross-section images presented were obtained after Au deposition. The distribution of the pillars reproduces the hexagonal close packed structure of the colloidal monolayer mask. An analysis of the FESEM images permitted an estimation on the evolution of the pillar geometry. The evolution of the width and height of the pillars is plotted in Figure 2f. In the range of the RIE times studied, the width of the Si pillars was relatively stable, until a critical drop was induced at circa 300 s. In the same period of time, the height increased at a rate which, remarkably, overpassed the lateral etching rate (a mean value of 2.21 nm/s versus −0.33 nm/s, respectively, in the 210, 280 s range). Similarly, after circa 300 s a drastic reduction and inversion of the in-depth etching rate was induced. This critical point is related to the consumption of the colloidal structures, which initiates a stage of overall etching in the Si structure that completely changes the pillar geometry leading to its consumption. For further Au sputtering processing, we considered the structures displayed by the RIE at 240 s. 

### 3.2. Deposition and Post-Processing of the Gold Layer

**Figure 3:**
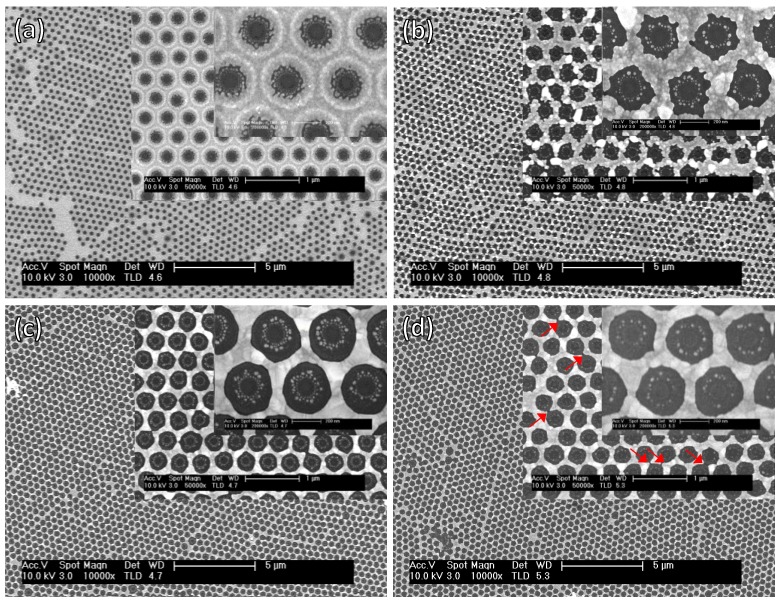
FESEM images at different magnifications of the surface structure of the Au film as deposited through the colloidal mask on Si (**a**), after KI immersion for 1 min (**b**), after annealing at 300 °C (**c**), and after both annealing and KI etching (**d**).

Au deposition on Si was performed by standard sputtering in an Ar discharge. The effect of different post-processing treatments was considered, looking both at the electronic properties of the processed Au layer (Si substrate) and the resulting optical properties (glass substrate). The main post-processing parameters were thermal annealing at 300 °C, which is known to induce the coalescence of bigger grains of Au, and wet etching in KI dilutions, which dissolves weakly bound Au atoms. The post-processing was always applied to samples after the lift-off of the colloidal monolayer. Figure 3 shows FESEM images at different magnifications on the surface structure of the Au film as deposited through the colloidal mask on Si, after KI immersion for 1 min, annealing at 300 °C, or after both annealing and KI etching (Figure 2a–d, respectively). 

Again, the Au film distribution effectively reproduces the colloidal monolayer and an Au ring still reminds the presence of the colloidal particles (See Figure 2a at different magnifications). However, the KI etching erases this ring structure and partially erodes the surface revealing a more granular film with irregularly shaped holes (Figure 2b). The thermally induced grain growth can be clearly identified in Figure 2c, leading to a partial dewetting of the hole area and a tendency towards polygonal shapes in the hole-edge. The dual effect of annealing and KI etching is evident in Figure 2d and is coherent with both the effect of grain growth induced by the annealing and the effect of highlighting the grain boundaries induced by KI. The dual effect is responsible of a film retraction, which results in observable Au discontinuities highlighted with red arrows in Figure 2d.

We searched for potential changes in the local structure of the Au sputtered cavity films by performing a XAS study before and after the different post-processing conditions. Au film properties are, in fact, extremely substrate dependent (granular growth, adherence, cluster convergence, etc.), so we used extended x-ray absorption fine structure (EXAFS) to get an insight into the nanostructure of the Au film. Since the multiple scattering paths have a greater degeneracy than the paths corresponding to single scattering events, only the last ones were taken into account in the present analysis. Therefore, the results corresponding to the fitting of the first four shells are shown in Table 1. Meanwhile, the data corresponding to their radial distribution functions (RDF) is shown in Figure 3a. The Au L3 edge region (around 11.9 keV) of the spectra for the different post-processing conditions did not show any particular differences compared to the sputtered samples, suggesting that there are no changes in its electronic structure and thus, there are no interactions with other atoms (the poor interaction between Si and Au is a well-known issue). All RDF show rather similar profiles up to circa 3.8 Å (corresponding to the third shell in the model used to fit the data, as seen in Table 1), which reflects the similar crystalline structure of the Au cavity films. However, despite the similarity in the first two shells, the high degeneracy of the coordination number in the third shell (N = 24) makes it possible to observe small perturbations in the structure through the Debye–Waller factor (σ^2^) for distances larger than 3.8 Å to the absorbing atom (see Table 1 and compare with Figure 3a).

This behavior of the RDF correlates with the exposure of the samples to KI. In fact, the oscillation starting from this critical value is higher in the samples exposed to KI. This is in agreement with the expected effect of KI, which is known to preferentially actuate on Au atoms standing on defects of Au (111) terraces. The KI is thus responsible for a surface cleaning that tends to increase the overall Au crystal order (reducing the Debye–Waller factor), which is reflected in the RDF. This effect of increased order is even more remarkable for the sample with dual annealing and KI etching.

**Figure 4:**
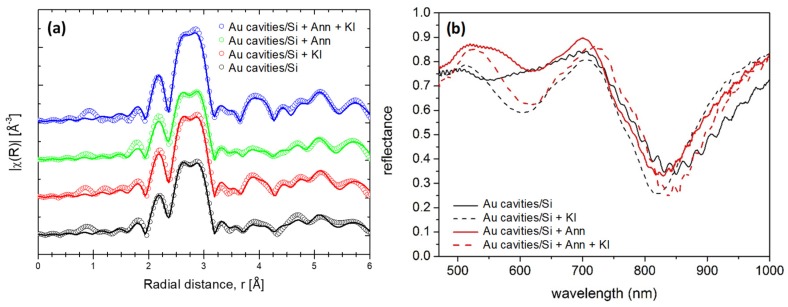
(**a**) Radial distribution functions for the Au films deposited through the colloidal mask on Si before and after post-processing by KI immersion for 1 min (KI), after annealing at 300 °C (Ann) and after both annealing and KI etching (Ann + KI). (**b**) Reflectance spectra of the Au cavity films deposited on SiO_2_ before and after post-processing in identical conditions as described in (**a**).

In order to determine the potential advantage of Au deposition/post-processing treatment, we performed optical reflectance measurements that could show some influence on the plasmonic response. The measurements were performed on Au nanocavity analogues deposited on glass, whose reflectance–transmittance behavior was already correlated in theoretical and experimental models [47]. The reflectance spectra of the Au cavity samples before and after post-processing in the 400–1000 nm range is presented in Figure 4b. The main trough of the spectra at circa 850 nm was attributed to an LSP, although these kind of surfaces are known to simultaneously support LSPs and SPPs, with the latter modes expected below 700 nm [47]. The comparison of the LSP mode at circa 850 nm for the different samples does not show a clear advantage of the treatments, even though the resonance was slightly narrowed after post-processing with KI solution (see dotted lines in the spectra of Figure 4b). In any case, for the forthcoming experiments, the studies were preliminarily performed with deposited Au, that is, including no post-processing. 

### 3.3. Optical Response of the Au/SiΠ Structures

**Figure 5:**
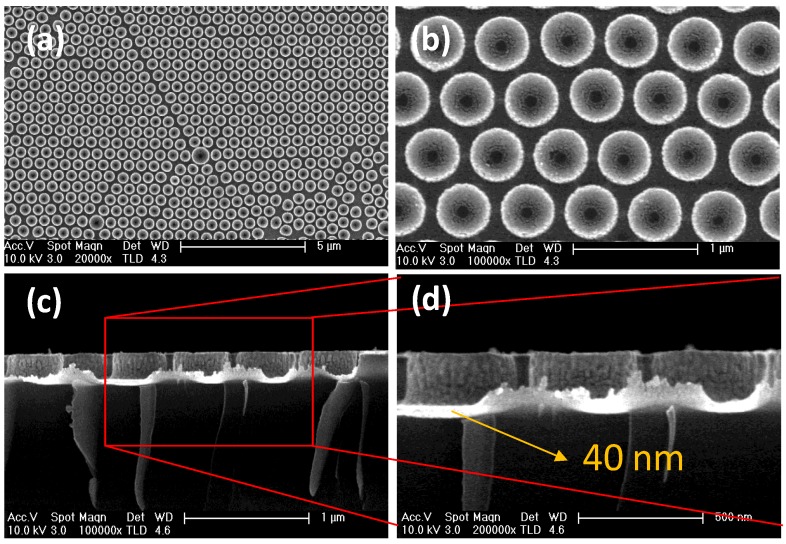
General FESEM view of the in plane order and presence of defects in Au/SiΠ structures formed after 240 s RIE and 280 s sputtering of Au with no further post-processing (**a**). Higher magnification image highlighting the absence of Au on the center and its presence on the lateral part (**b**). Cross section view confirming the Au lateral coating (**c**) and a higher magnification (**d**) providing mean thickness at the base and details of the columnar morphology at the pillar edge.

The final morphology of the Au/SiΠ structures grown after 240 s of RIE processing, and 280 s of Au sputtering are shown in Figure 5. The top-view FESEM image in Figure 5a illustrates the ordered surface distribution of the pillars, presenting characteristic defects such as dislocations induced by bigger substitutional pillars. The higher magnification image (Figure 5b) demonstrates that the center of the pillar is an open window facing the Si at the center of the pillars in the environment. The cross-section view in Figure 5c demonstrates that the Au layer is in fact conformal and covers the pillar perimeter and the interconnecting valleys of the pillars. The higher magnification image shows that the Au layer presented some thickness distribution with a mean thickness estimated at 40 nm. The coating gives rise, as shown in Figure 5d, to columnar structures of Au around the Si pillar. 

**Figure 6:**
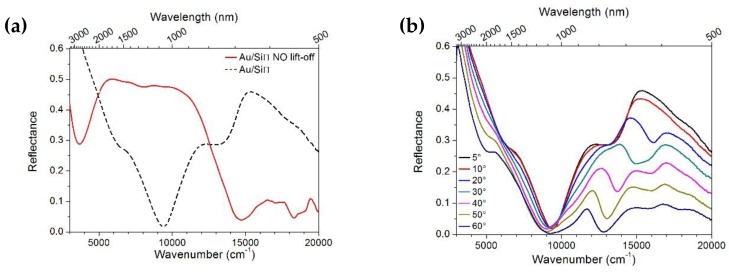
(**a**) Reflectance measurements in the 500–3300 nm range at near-normal incidence of the Au/SiΠ structures before (red line) and after (black line) colloidal mask lift-off; (**b**) Angle dependent reflectance for the Au/SiΠ structures after lift-off of TM light polarization.

The reflectance response of the Au/SiΠ system was evaluated at near normal incidence in the 3300–500 nm wavelength range before and after application of the critical lift-off process, as shown in Figure 6a. The response of the system is clearly linked to this latter process and clearly reflects a change in the active window for the Au/SiΠ structures prior to and after the lift-off process (the red line and black line, respectively). The response of the sample studied prior to the lift-off process presented a dominantly absorbing behavior (below 10% reflectance) in the visible range 700–500 nm, which is typical of corrugated Au surfaces [48]. 

On the other hand, after the lift-off process the spectral response was somehow complementary to the one observed before lift-off. The higher absorption was observed in the near infrared (NIR) range, with a clear trough at circa 1100 nm. At lower wavelengths the curves presented higher reflectance than those previous. Figure 6b shows the reflectance spectra of the Au/SiΠ structure (after lift-off) for transverse magnetic field (TM) polarization and variable incident angle. A minimum is evident around 1100 nm while other dispersive features are clearly observable in the visible spectral region at around 600–800 nm. Since interest has arisen in using these type of samples for SERS application, we performed a deeper investigation in the NIR-vis spectral region. 

**Figure 7:**
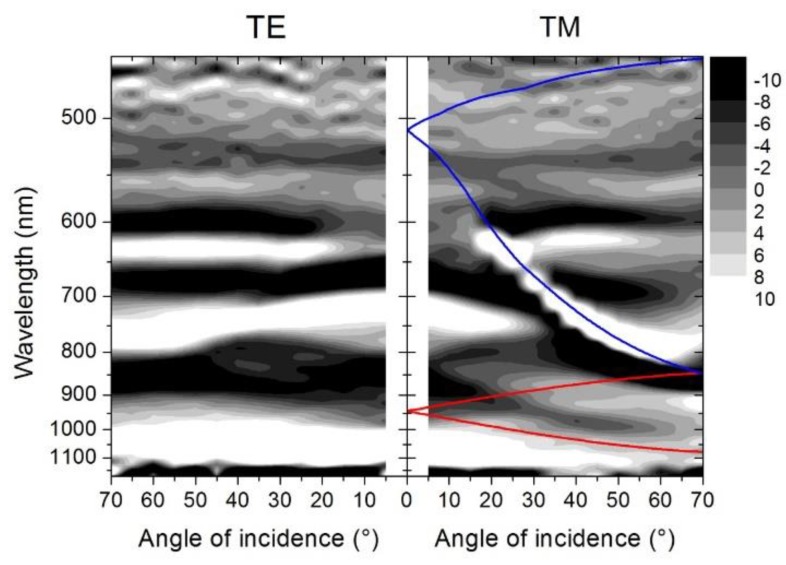
Dispersion map of the second derivative of the reflectance of the TE and TM light polarization in the final Au/SiΠ structure.

In order to interpret the optical response of the final Au/SiΠ structure (after lift-off) we have plotted the second derivative of the reflectance as a function of the incident angle for transeverse electric field (TE) and TM polarized light (see Figure 7). The TE map highlights some reflectance minima (bright area) around 640 nm, 770 nm, and 1050 nm. These reflectance minima corresponded to localized plasmon resonance (LSP) that, in TE polarization, are clearly evident with no dispersion. The LSP were excited in the silicon pillar and in the apertures on the top of the pillars. 

In the TM map, a more complicated behavior could be observed instead. At small incident angles, the two LSP at 770 nm and 1050 nm were already visible while the LSP at 640 nm was more visible at large incident angles. In TM, the LSPs modes were clearly influenced by the excitation dispersive modes. The coloured lines superimposed on the TM map are the theoretical calculations of the surface plasmon polaritons (SPPs) at the Au-silicon and Au-air interface (red line and blue line respectively) folded by the presence of the lattice periodicity on the sample surface.

The second order SPP in the Au-Si interface was evident in the map at around 950 nm, and caused the dispersion of the LSP modes active in the same spectral region. The first order is instead evident in the spectrum (Figure 6b) as a dispersive minimum at around 550 nm. In the VIS, the SPP at the Au-air interface strongly interacted with the LSP modes. At around 800 nm, an anti-crossing between the SPP at the two interfaces could be observed. In conclusion, the map in TM polarization highlights a very complex interaction pattern between LSPs and SPPs, with the rising of hybrid modes in the system.

### 3.4. SERS Performance

The main trough of the reflectance spectra Au/SiΠ structures suggests that there are potential possibilities to create hot-spots in the structures in the NIR-vis range. This observation led us to consider the possibility of using these structures for SERS applications. We started by exploring the SERS enhancement in the acquisition of spectra of two typical dyes: Malachite Green and Rhodamine 6G. After the optimization of the acquisition conditions for each of the dyes, the SERS spectra were obtained at three different excitation wavelengths. 

**Figure 8:**
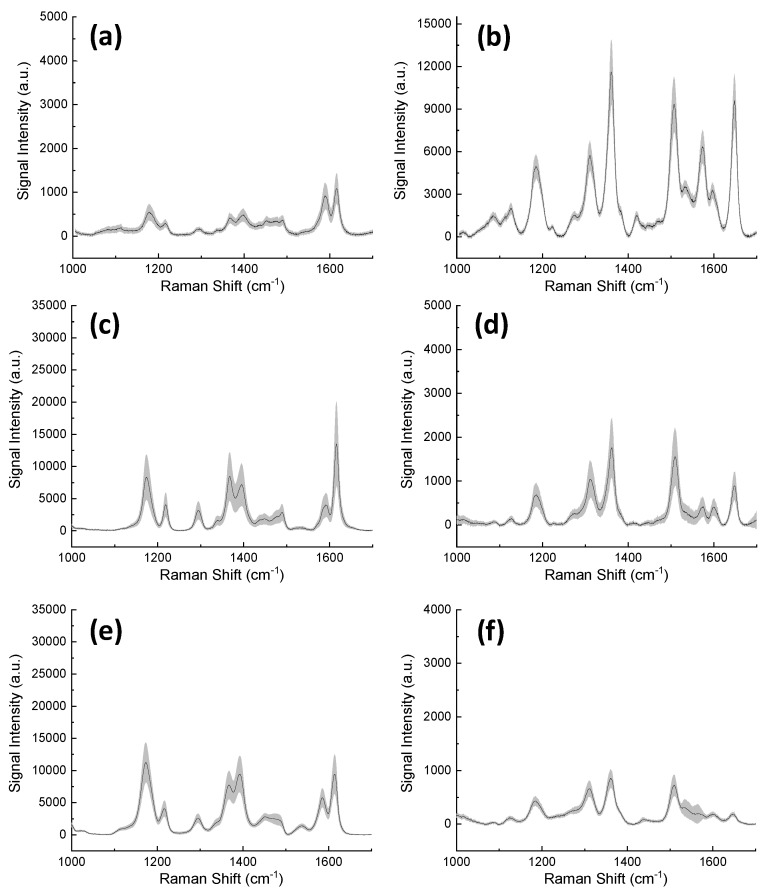
Raman analysis with Au/SiΠ structures of 2 μM Malachite Green (**a**-**c**-**e**) and 2 μM Rhodamine 6G (**b**-**d**-**f**) using three different excitation lasers 533 nm (**a**,**b**), 633 nm (**c**,**d**), and 785 nm (**e**,**f**).

The SERS spectra are shown in Figure 8, with MG and R6G spectra shown at the left (a,c,e) and right (b,d,f), respectively, with excitation at 533, 633 and, 785 nm, from top to bottom. For MG at 2 μM we could confirm that the excitation at lower wavelength was responsible for inducing a preferential SERS enhancement of the characteristic bands at higher wavenumbers. To illustrate this, we traced the relative intensity of the 1620 cm^−1^ band with respect to the 1170 cm^−1^ band for the three laser excitations, and one can notice a clear decrease of the band intensity ratio as the laser wavelength increases. The same observation is valid for the analyses of R6G at 2 μM, taking for instance the characteristic bands of R6G at 1650 and 1185 cm^−1^. Notably, while the spectrum of MG was acquired with increased resolution of the 785 nm excitation, the best resolution for R6G spectra is obtained with 533 nm excitation. This serves as a reminder that the optimization of a SERS substrate implies consideration of a specific molecule and a particular excitation wavelength.

**Figure 9:**
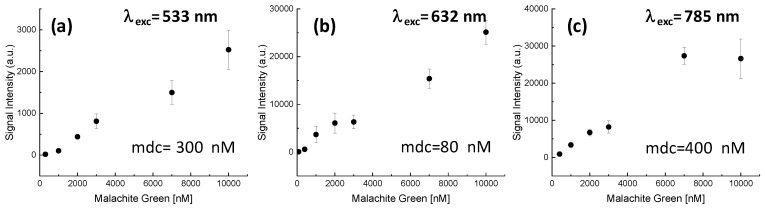
Calibration of the intensity of the Malachite Green band at 1170 cm^−1^ for different concentrations in the 16–10000 nM range for excitation with 533 (**a**), 633 (**b**), and 785 (**c**) nm lasers and details of the corresponding limit of detection.

We considered studying the SERS enhancement effect further at different concentrations, using the MG molecule as a model and its band at 1170 cm^−1^ as a reference. The results for the different excitation wavelengths are presented in Figure 9. For the excitations at 533 and 633 nm, it can be concluded that, in the 16–10000 nM concentrations range, the enhancement effect is linear. However, for the excitation at 785 nm, the enhancement effect presented a saturation at the highest concentration. The plot allows the determination of the minimum detectable concentration (mdc) in each of these configurations, being 300 nM, 80 nM, and 400 nM for excitation and 533, 633, and 785 nm lasers, respectively. This kind of calibration of the SERS substrates is necessary to extract the characteristic Raman pattern of a molecule from a complex sample with competing molecules.

**Figure 10:**
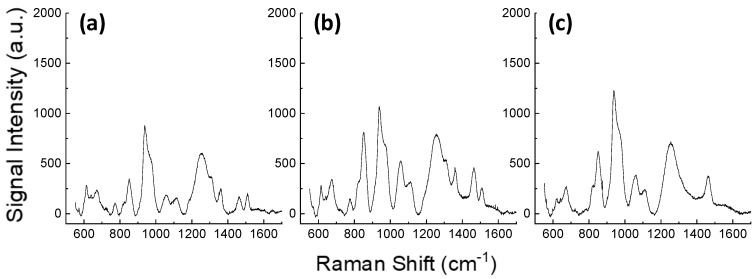
SERS analyses with Au/SiΠ structures of blood plasma filtered at 3, 10, and 30 KDa (**a**–**c**, respectively) aiming at characterization of small biomolecules.

We then considered using the Au/SiΠ structures to analyse complex biomolecular media, such as blood plasma after filtering the different molecular weights of the contained proteins. These analyses were performed using the 785 nm excitation to avoid the auto-fluorescence of the three aromatic amino acids (phenylalanine, tyrosine, and tryptophan) as much as possible. The results of the SERS analyses are shown in Figure 10, and they highlight the importance of a controlled sieving process in the original blood plasma. This process facilitates adhesion to the surface of the small metabolites presenting the narrow Raman bands that are of interest in biomedical SERS studies [49]. The spectra shown in Figure 10, in fact, allow for the identification of an increase in the resolution of the spectra for the decreased molecular weight of the biomolecules remaining in the blood plasma. Note, for instance, how the band at circa 1480 cm^−1^ in the 30 KDa filtered blood plasma (Figure 10c), is resolved with two modes (1480 and 1510 cm^−1^) after filtering at 10 KDa (Figure 10b), and even with better band resolution after sieving at 3 KDa (Figure 10a). This SERS effect is related to the homogeneity of the evanescent electromagnetic field at the biomolecule–plasmonic structure interface [48]. For bigger adsorbed molecules, such as proteins, the interacting field was more distributed, resulting in the widening of the band interactions. In our system, the results suggest that even if the effect of resolution is patent, the Au/SiΠ structures may be effective in the well-resolved characterization of biomolecules attaining sizes of several tens of KDa. 

## 4. Conclusions

Two different plasma processes (reactive ion etching and plasma sputtering) were used sequentially to fabricate Au/SiΠ structures. The process implies a previous step of self-assembly of a colloidal monolayer, which is followed by the reactive ion etching process. We saw that pillars of different aspect ratios can be fabricated by experimenting with plasma parameters, such as plasma exposure. We also saw that the aspect ratio of the structures increased with time as long as the polystyrene colloids were not consumed, which drastically erodes the formed pillar structures. We further studied the plasma sputtering deposition of Au and post-processing methods onto the pillar structures. We noticed that, even though the wet etching of Au in mild KI tended to promote the presence of more ordered Au clusters, the influence in the spectral response was only moderate. 

We further showed that the optical properties of the processed structures drastically changed before and after the lift-off of the polystyrene bead monolayer. The dependence of the observed modes with the angle of incidence of light suggested the presence of both SPP and LSP modes, with a remarkable 0% reflectance band in the NIR range created when the Au/SiΠ structures were formed. 

The structures were further used as SERS supports for two model molecules. It was confirmed that the SERS spectra depend on resolution and relative intensity in the excitation wavelength. The dependence of the amplification with the concentration of the dye can be considered linear in the first approximation. We then confirmed that the structures are of potential interest for the well-resolved analysis of biomolecules with molecular weight in the range of tens of KDa. 

## Figures and Tables

**Table 1:** Results from the EXAFS fittings of the different Au cavities (N represents the coordination number, R the distance to the absorbing atom, and σ^2^ is the shell mean-square disorder). All shells correspond to Au-Au single scattering events.

	Sample	Au Cavities/Si + Ann + KI	Au Cavities/Si + Ann	Au Cavities/Si + KI	Au Cavities/Si
Shell 1	N	12	12	12	12
	R (Å)	2.88 ± 0.01	2.87 ± 0.01	2.87 ± 0.01	2.87 ± 0.01
	σ^2^(×10^−4^) (Å^2^)	27 ± 1	39 ± 2	31 ± 2	36 ± 2
Shell 2	N	6	6	6	6
	R (Å)	4.08 ± 0.02	4.06 ± 0.02	4.06 ± 0.02	4.06 ± 0.02
	σ^2^(×10^−4^) (Å^2^)	28 ± 6	76 ± 20	40 ± 13	71 ± 20
Shell 3	N	24	24	24	24
	R (Å)	4.99 ± 0.03	4.97 ± 0.02	4.97 ± 0.03	4.97 ± 0.02
	σ^2^(×10^−4^) (Å^2^)	43 ± 5	68 ± 9	49 ± 7	64 ± 9
Shell 4	N	12	12	12	12
	R (Å)	5.77 ± 0.03	5.74 ± 0.03	5.76 ± 0.03	5.74 ± 0.03
	σ^2^(×10^−4^) (Å^2^)	26 ± 11	25 ± 10	36 ± 17	57 ± 26

## References

[1] Jani A.M.M., Losic D., Voelcker N.H. (2013). Nanoporous anodic aluminium oxide: Advances in surface engineering and emerging applications. Prog. Mater. Sci..

[2] Ding C.Q., Zhu A.W., Tian Y. (2014). Functional Surface Engineering of C-Dots for Fluorescent Biosensing and in Vivo Bioimaging. Acc. Chem. Res..

[3] Yao B., Zhang J., Fan X.L., He J.P., Li Y. (2019). Surface Engineering of Nanomaterials for Photo-Electrochemical Water Splitting. Small.

[4] Placido T., Tognaccini L., Howes B.D., Montrone A., Laquintana V., Comparelli R., Curri M.L., Smulevich G., Agostiano A. (2018). Surface Engineering of Gold Nanorods for Cytochrome c Bioconjugation: An Effective Strategy To Preserve the Protein Structure. Acs Omega.

[5] Wang D., Hou S.C., Wu H.W., Zhang C., Chu Z.Z., Zou D.C. (2011). Fiber-shaped all-solid state dye sensitized solar cell with remarkably enhanced performance via substrate surface engineering and TiO_2_ film modification. J. Mater. Chem..

[6] Pellacani P., Fornasari L., Rodriguez C., Torres-Costa V., Marabelli F., Silvan M.M. (2019). Porous Silicon Bragg Reflector and 2D Gold-Polymer Nanograting: A Route Towards a Hybrid Optoplasmonic Platform. Nanomaterials.

[7] Gandhiraman R.P., Volcke C., Gubala V., Doyle C., Basabe-Desmonts L., Dotzler C., Toney M.F., Iacono M., Nooney R.I., Daniels S. (2010). High efficiency amine functionalization of cycloolefin polymer surfaces for biodiagnostics. J. Mater. Chem..

[8] Arroyo-Hernandez M., Manso-Silvan M., Lopez-Elvira E., Munoz A., Climent A., Martinez Duart J.M. (2007). One step processing of aminofunctionalized gate oxides. Biosens. Bioelectron..

[9] Kim J.H., Lee M.J., Park E.J., Lee J.Y., Lee C.J., Min N.K. (2012). Plasma Processing: Technology for the Batch Fabrication of Carbon Nanotube Film Electrodes for Biointerfaces. Plasma Process. Polym..

[10] Flynn C.N., Byrne C.P., Meenan B.J. (2013). Surface modification of cellulose via atmospheric pressure plasma processing in air and ammonia-nitrogen gas. Surf. Coat. Technol..

[11] Fisher E.R. (2013). Challenges in the Characterization of Plasma-Processed Three-Dimensional Polymeric Scaffolds for Biomedical Applications. Acs Appl. Mater. Interfaces.

[12] Chichti E., Henrion G., Cleymand F., Jamshidian M., Linder M., Arab-Tehrany E. (2013). Effects of Ar-N-2-O-2 Microwave Plasma on Poly-L-Lactic Acid Thin Films Designed for Tissue Engineering. Plasma Process. Polym..

[13] Borges A.M.G., Benetoli L.O., Licinio M.A., Zoldan V.C., Santos-Silva M.C., Assreuy J., Pasa A.A., Debacher N.A., Soldi V. (2013). Polymer films with surfaces unmodified and modified by non-thermal plasma as new substrates for cell adhesion. Mater. Sci. Eng. C-Mater. Biol. Appl..

[14] Formosa F., Sanchez-Vaquero V., Rodriguez-Navas C., Munoz-Noval A., Tejera-Sanchez N., Silvan M.M., Garcia-Ruiz J.P., Marletta G. (2010). Evaluation of Plasma Modified Polycaprolactone Honeycomb Scaffolds by Human Mesenchymal Stem Cells Cultured in Vitamin D Differentiation Medium. Plasma Process. Polym..

[15] Sheng L.M., Luo M., Sun X.J., Lin N.M., Mao W.M., Su D. (2013). Serum fibrinogen is an independent prognostic factor in operable nonsmall cell lung cancer. Int. J. Cancer.

[16] Seo H., Ichida D., Hashimoto S., Uchida G., Itagaki N., Koga K., Shiratani M. (2015). Photovoltaic application of Si nanoparticles fabricated by multihollow plasma discharge CVD: Dye and Si co-sensitized solar cells. Jpn. J. Appl. Phys..

[17] Ramadan R., Abdelhady K., Manso-Silvan M., Torres-Costa V., Martin-Palma R.J. (2019). Microwave plasma and rapid thermal processing of indium-tin oxide thin films for enhancing their performance as transparent electrodes. J. Photonics Energy.

[18] Chen C.C., Bai H.L., Chang S.M., Chang C.L., Den W. (2007). Preparation of N-doped TiO2 photocatalyst by atmospheric pressure plasma process for VOCs decomposition under UV and visible light sources. J. Nanoparticle Res..

[19] Zhang X.L., Zhang L.J., Li Y.C., Di L.B. (2015). Atmospheric-pressure cold plasma for fabrication of anatase rutile mixed TiO_2_ with the assistance of ionic liquid. Catal. Today.

[20] Sexton B.A., Feltis B.N., Davis T.J. (2008). Characterisation of gold surface plasmon resonance sensor substrates. Sens. Actuators A-Phys..

[21] Ince R., Narayanaswamy R. (2006). Analysis of the performance of interferometry, surface plasmon resonance and luminescence as biosensors and chemosensors. Anal. Chim. Acta.

[22] Schell N., Jensen T., Petersen J.H., Andreasen K.P., Bottiger J., Chevallier J. (2003). The nanostructure evolution during and after magnetron deposition of Au films. Thin Solid Film..

[23] Peto G., Molnar G.L., Paszti Z., Geszti O., Beck A., Guczi L. (2002). Electronic structure of gold nanoparticles deposited on SiOx/Si(100). Mater. Sci. Eng. C-Biomim. Supramol. Syst..

[24] Picciolini S., Mehn D., Morasso C., Vanna R., Bedoni M., Pellacani P., Marchesini G., Valsesia A., Prosperi D., Tresoldi C. (2014). Polymer Nanopillar - Gold Arrays as Surface-Enhanced Raman Spectroscopy Substrate for the Simultaneous Detection of Multiple Genes. Acs Nano..

[25] Bally M., Halter M., Voros J., Grandin H.M. (2006). Optical microarray biosensing techniques. Surf. Interface Anal..

[26] Parracino M., Pellacani P., Colpo P., Ceccone G., Valsesia A., Rossi F., Silvan M.M. (2016). Biofouling Properties of Nitroxide-Modified Amorphous Carbon Surfaces. Acs Biomater. Sci. Eng..

[27] Chen Z.Q., Liu M.H., Tang L., Lv J.H., Wen Y.F., Hu X.W. (2009). Production of planar-type overdense plasma with resonant excitation of surface plasmon polaritons. J. Appl. Phys..

[28] Li M.W., Qiu Y.Y., Fan C.C., Cui K., Zhang Y.M., Xiao Z.Y. (2018). Design of SERS nanoprobes for Raman imaging: Materials, critical factors and architectures. Acta Pharm. Sin. B.

[29] Li Y.S., Church J.S. (2014). Raman spectroscopy in the analysis of food and pharmaceutical nanomaterials. J. Food Drug Anal..

[30] Fukami K., Chourou M.L., Miyagawa R., Munoz Noval A., Sakka T., Manso-Silvan M., Martin-Palma R.J., Ogata Y.H. (2011). Gold Nanostructures for Surface-Enhanced Raman Spectroscopy, Prepared by Electrodeposition in Porous Silicon. Materials.

[31] Zong C., Xu M.X., Xu L.J., Wei T., Ma X., Zheng X.S., Hu R., Ren B. (2018). Surface-Enhanced Raman Spectroscopy for Bioanalysis: Reliability and Challenges. Chem. Rev..

[32] Gong Z., Ji J., Wang J. (2019). Photocatalytic Reversible Reactions Driven by Localized Surface Plasmon Resonance. Catalysts.

[33] Shen Y., Miao P., Hu C., Wu J., Gao M., Xu P. (2018). SERS-Based Plasmon-Driven Reaction and Molecule Detection on a Single Ag@MoS2 Microsphere: Effect of Thickness and Crystallinity of MoS2. Chemcatchem.

[34] Pellacani P., Torres-Costa V., Agullo-Rueda F., Vanna R., Morasso C., Manso Silvan M. (2019). Laser writing of nanostructured silicon arrays for the SERS detection of biomolecules with inhibited oxidation. Colloids Surf. B-Biointerfaces.

[35] Silvan M.M., Hernandez M.A., Costa V.T., Palma R.J.M., Duart J.M.M. (2006). Structured porous silicon sub-micrometer wells grown by colloidal lithography. Europhys. Lett..

[36] Tsai P.S., Yang Y.M., Lee Y.L. (2006). Fabrication of hydrophobic surfaces by coupling of Langmuir-Blodgett deposition and a self-assembled monolayer. Langmuir.

[37] Mizeikis V., Juodkazis S., Ye J.Y., Rode A., Matsuo S., Misawa H. (2003). Silicon surface processing techniques for micro-systems fabrication. Thin Solid Film..

[38] Men D.D., Wu Y.Y., Wang C., Xiang J.H., Yang G.L., Wan C.J., Zhang H.H. (2018). Wafer-Scale Hierarchical Nanopillar Arrays Based on Au Masks and Reactive Ion Etching for Effective 3D SERS Substrate. Materials.

[39] Xia J., Tominaga R., Fukamitsu S., Usami N., Shiraki Y. (2009). Generation and Wavelength Control of Resonant Luminescence from Silicon Photonic Crystal Microcavities with Ge Dots. Jpn. J. Appl. Phys..

[40] Hamza S., Ignaszak A., Kiani A. (2017). Synthesis of Electrical Conductive Silica Nanofiber/Gold Nanoparticle Composite by Laser Pulses and Sputtering Technique. Nanoscale Res. Lett..

[41] Kaisar N., Huang Y.T., Jou S., Kuo H.F., Huang B.R., Chen C.C., Hsieh Y.F., Chung Y.C. (2018). Surface-enhanced Raman scattering substrates of flat and wrinkly titanium nitride thin films by sputter deposition. Surf. Coat. Technol..

[42] Kinner L., Bauch M., Wibowo R.A., Ligorio G., List-Kratochvil E.J.W., Dimopoulos T. (2019). Polymer interlayers on flexible PET substrates enabling ultra-high performance, ITO-free dielectric/ metal/ dielectric transparent electrode. Mater. Des..

[43] Pandit P., Schwartzkopf M., Rothkirch A., Roth S.V., Bernstorff S., Gupta A. (2019). Structure-Function Correlations in Sputter Deposited Gold/Fluorocarbon Multilayers for Tuning Optical Response. Nanomaterials.

[44] Ellmer K. (2000). Magnetron sputtering of transparent conductive zinc oxide: Relation between the sputtering parameters and the electronic properties. J. Phys. D-Appl. Phys..

[45] Rodriguez C., Pellacani P., Manso-Silvan M. (2019). Organo-Silane Self-Assembly on Porous Silicon and Silica Particle based Sensors. World Sci. Ref. Hybrid Mater..

[46] Ravel B., Newville M. (2005). Athena, Artemis, Hephaestus: Data analysis for X-ray absorption spectroscopy using IFEFFIT. J. Synchrotron Radiat..

[47] Giudicatti S., Valsesia A., Marabelli F., Colpo P., Rossi F. (2010). Plasmonic resonances in nanostructured gold/polymer surfaces by colloidal lithography. Phys. Status Solidi A-Appl. Mater. Sci..

[48] Martynova N.A., Goldt A.E., Maslakov K.I., Savilov S.V., Grigorieva A.V. (2018). Electroplating of porous gold films for SERS analysis of heme derivatives. J. Mater. Sci..

[49] Lin D., Pan J.J., Huang H., Chen G.N., Qiu S.F., Shi H., Chen W.W., Yu Y., Feng S.Y., Chen R. (2014). Label-free blood plasma test based on surface-enhanced Raman scattering for tumor stages detection in nasopharyngeal cancer. Sci. Rep..

